# Narcissistic coaches and athletes’ individual rowing performance

**DOI:** 10.1038/s41598-023-48239-6

**Published:** 2023-11-29

**Authors:** Barbara Nevicka, Emma J. G. van Gerven, Constantine Sedikides

**Affiliations:** 1 Department of Psychology, University of Amsterdam, Amsterdam, The Netherlands; 2 Amsterdam Business School, University of Amsterdam, Amsterdam, The Netherlands; 3 School of Psychology, University of Southampton, Southampton, UK

**Keywords:** Human behaviour, Psychology

## Abstract

Narcissism, a personality trait marked by an excessively self-aggrandizing, entitled, and dominant orientation, has been associated with high performance under competitive pressure, as these contexts afford the opportunity to self-enhance. Narcissism is often characteristic of organizational and political leaders, yet little is known about narcissism in sports coaches. We propose that in a competitive context narcissistic coaches could inspire and motivate their athletes to raise their performance. We investigated the association between coach narcissism and athletes’ performance, and the role of athletes’ perceived self-enhancement opportunity as a potential mediating mechanism. We examined coach narcissism, athletes’ individual end times (i.e., performance), and athletes’ perceptions of self-enhancement opportunity during annual national indoor rowing competitions in 266 national level competitive rowers from 52 rowing clubs. Results of multilevel analyses showed that coach narcissism positively predicted athlete performance, and this was explained by athletes’ perceived opportunity to self-enhance during the competition. Thus, narcissistic coaches seem to reinforce athletes’ perceptions that competition provides them with an opportunity to show off their skills, which in turn accounts for athletes’ better performance in comparison to athletes who do not train with narcissistic coaches. The findings point to a potentially functional side of narcissism in coaching.

## Introduction

Among the many factors that impact athletes’ motivation and subsequent performance, coaches’ behavior is particularly important^1^. The relationship between athletes and their coaches is a central feature of an athlete's sport experience. For example, coaches stimulate athletes to set goals and teach them how to be strong physically and mentally^2^. Therefore, having a good coach can make a sizeable difference to how an athlete performs. In this article, we investigate how coaches’ personality conduces to athletes’ performance by focusing on the role of narcissism.

Narcissism (specifically, grandiose agentic narcissism), is a personality trait defined by a cognitive and affective preoccupation with oneself, dominance, and a belief that one is superior to others and entitled^3,4^. Narcissists often seek leadership positions to fuel their need for power and status^5,6^. Indeed, they possess many characteristics associated with leadership, such as extraversion and authority, which explains, in part, why they are likely to be promoted^7^ and why they emerge as leaders in groups^7–9^. Given narcissists’ likelihood of attaining leadership positions, narcissism should be considered a prototypical leader trait^10^ in the sports domain. Leadership is an essential element of coaching^1^, yet little is known about the relation between coaches’ narcissism and athletic performance. The scarce literature shows that narcissistic coaches adopt a controlling (vs. autonomy-supportive) coaching style^11,12^, and this is associated with athletes’ positive attitudes toward doping^13^.

The current study is the first to examine directly the link between coach narcissism and athlete’s performance. Paradoxically, narcissists possess both positive (e.g., confidence, extraversion, self-esteem, charm) and negative (e.g., disdain, exploitativeness, aggression, empathy deficits) characteristics^3,6^. Thus, it is not surprising that prior research on the effectiveness of narcissistic leaders has produced mixed findings, with a recent meta-analysis reporting a curvilinear relation^8^. This statistical pattern signals the relevance of contextual moderators, such as the temporality of observers’ perceptions (with narcissistic leaders viewed positively initially but then more negatively over time^14^), uncertainty (with narcissistic leaders viewed as more effective in uncertain situations^15^), and humility (with narcissistic leaders resorting to humility as a self-presentational tactic to placate their unfavorable sides^16^). In the sporting domain, we propose that a key contextual variable capable of harnessing a narcissistic leader’s (i.e., coach’s) beneficial side is competition.

Research to date has predominantly focused on the negative side of coach narcissism, primarily stemming from their controlling (i.e., authoritarian and pressuring) interpersonal style. Indeed, narcissistic coaches exhibit more controlling and less autonomy-supportive interpersonal styles, which is explained by their lower empathy^11^. A controlling interpersonal style can be damaging to athletes’ psychological well-being, while hindering their capacity for self-regulation and intrinsic motivation^17^. Narcissistic coach’s controlling style was found to be related to athletes’ lower need (i.e., autonomy, competence, relatedness) satisfaction and more positive attitudes about doping^13^. Relatedly, the controlling style of narcissistic coaches predicts their moral disengagement, an antecedent of antisocial sport behavior^12^.

Although first indicators of the role of narcissistic coaches appear to point in the negative direction, prior research on narcissistic coaches did not take the context of competition into account when examining their influence on athletes nor did it examine athlete performance. Here, we propose that narcissistic coaches can be beneficial for athletes’ performance in competitive settings. Narcissists perform well in challenging or high-pressure contexts, such as a sport competition^16^, because such contexts enable them to show off and afford them opportunity for glory^18,19^. Narcissistic leaders can be charismatic, inspiring^20^, mentally tough^21^, and persistent in the face of obstacles^6^. As such, we would expect narcissistic coaches to be particularly eager to use their charismatic and inspirational communication (aspects of transformational leadership^22^) for motivating their athletes, thereby enhancing performance^23^ during competition.

Our hypothesis further relies on literature suggesting that narcissistic leaders are likely to merge their group’s and own identity, and experience psychological ownership^24,25^. Hence, narcissistic coaches might be likely to merge their team’s identity with their personal identity, particularly in competitive contexts. In such contexts, they might see an opportunity to self-enhance via their team (i.e., vicarious self-enhancement^26^), given that, as coaches, they cannot directly perform themselves. Consequently, the apparent alignment between self-enhancing goals and team goals^27^ would probably prompt them to perceive their athletes’ performance as reflective of their own performance. This, in turn, would stimulate narcissistic coaches to motivate their athletes to, similarly as themselves, construe the competitive context as an opportunity to self-enhance and showcase their skills, pursue glory, and win their competition. Indeed, when narcissistic CEOs experience higher organizational identification, they improve team processes and thereby organizational performance^27^. Therefore, we would expect athletes with narcissistic coaches to perceive competitive contexts as an opportunity to self-enhance and show off their skills to a greater extent than athletes with coaches lower on narcissism. Given that task-relevant self-enhancement can facilitate subsequent task performance due to higher self-efficacy beliefs that one can achieve their goals, stronger motivation, and persistence^28–31^, we propose that athletes’ greater perceptions of self-enhancement opportunity will account for the expected link between coach narcissism and athlete performance.

To summarize, (i) narcissistic leaders can be inspirational, thereby motivating for their athletes, and (ii) narcissism is linked with higher motivation and performance in contexts that provide opportunity for glory, such as competitions. In order to attain reflected glory via their team, narcissistic coaches would likely spur on their athletes to excel in competitive contexts by encouraging them to perceive such contexts as opportunity for self-enhancement and thereby helping boost their self-efficacy, motivation, and persistence. We thus hypothesize that coach narcissism is positively associated with athlete performance, and that this association is accounted for—at least in part—by athletes’ perceptions of self-enhancement opportunity. We tested these hypotheses assessing rowers’ performance during a prestigious annual competition.

### Exploring the role of athlete narcissism

Additionally, we explored whether narcissistic (vs. non-narcissistic) athletes are more or less strongly influenced by narcissistic coaches. Based on similarity-attraction theory^32,33^, which posits that people are attracted to and develop better relationships with others who are similar to them, athletes with higher narcissism might be more receptive to narcissistic coaches, also due to their own stronger self-enhancement needs, especially in competitive contexts, and this receptivity might be further amplified by such coaches. Narcissistic individuals have a high achievement motivation and are more driven to succeed^3,34^; as such, they might be more willing to invite, and embrace being coached by, a narcissistic coach. In support of these possibilities, teacher and student narcissism congruence predict higher academic course grades^35^, and narcissistic managers form more favorable impressions of employees who exhibit behavior consistent with narcissism, namely self-promotion^36^. On the other hand, dominance-complementarity theory^37,38^ suggests that matching the dominance and assertiveness of one interaction partner with submissiveness and compliance of another culminates in more harmony and optimal social interactions. Given that narcissism is characterized by dominant behavior and power strivings^6,11,39^, narcissistic coaches and narcissistic athletes may experience greater friction and conflict, thus impeding narcissistic coach’s influence on such athletes. Consistent with this possibility, supervisor-subordinate relationship conflict is higher when narcissistic leaders work with more dominant employees^40^. Consequently, given that both positive and negative effects are plausible, we wondered about the role of athlete narcissism in influencing the relation between coach narcissism and athlete performance.

## Methods

### Participants and procedure

We collected data from rowers who competed in the Dutch National Indoor Rowing Championships across 4 years. This competition, which takes place annually, is well-known and prestigious, and involves individual athletes rowing solo for 2000 m on indoor rowing machines. There is a large audience present, a live tracker (also online), newspaper photographers, and members of the media (camera crew and presenters) who report on the competition. We contacted the Board of Directors from Dutch rowing clubs asking them to share our questionnaire link with all competing rowers. The final sample consisted of 266 national level competitive rowers, including some professional rowers, (50.8% women, 49.2% men; *M*_age_ = 21.34, *SD* = 4.49) from 52 rowing clubs, trained by 158 coaches (76.3% men, 23.7% women). Participants had, on average, 2.87 years (*SD* = 2.71) of rowing experience and had trained with their coach for 1.01 years (*SD* = 1.20). The online questionnaire was available one week in advance of the competition. Participation was voluntary and confidential, and informed consent was obtained from all participants. The study protocol was approved by the Institutional Ethics Review Board of the University of Amsterdam, and all research was carried out in accordance with relevant guidelines/regulations.

### Measures

#### Coach narcissism

We used the 16-item Narcissistic Personality Inventory (NPI^41^) to assess athletes’ perceptions of coach narcissism^42^. Observers are fairly accurate in detecting others’ grandiose narcissism^43–46^. Participants chose between narcissistic (“My coach thinks that he/she is a special person”; coded as 1) and non-narcissistic (“My coach thinks that he/she is no better or worse than most people”; coded as 0) statements. We computed the NPI score as the average of the 16 items (*M* = 0.44, *SD* = 0.23, α = 0.79). The intraclass correlation coefficients showed good interrater reliability (ICC1 = 0.35, ICC2 = 0.48, *F*(108, 157) = 1.93, *p* < 0.001). Interrater agreement was likewise shown to be strong (r_wg_ = 0.98)^47^.

#### Individual athlete performance

Participants’ individual end times (m:ss.ss) were recorded and made available on the official website of the Dutch National Indoor Championships competition. We used these end times as indicators of performance (*M* = 07:07.86, *SD* = 00:35.73).

#### Athlete perceived self-enhancement opportunity

We assessed perceived self-enhancement opportunity with five items that we constructed for the purposes of this research. To do so, we relied on the conceptual definition of situational self-enhancement opportunity, which “denotes the degree to which one can potentially win glory by performing well” (p. 820^19^). The items were: “A good performance in the championship will lead others to admire me”, “A good performance in the championship will ensure that others respect me”, “During the championship I can distinguish myself”, “A good performance in the championship will lead me to get status”, “During the championship I can showcase my skills” (1 = *strongly disagree*, 7 = *strongly agree*). We averaged responses to form the relevant index (*M* = 4.94, *SD* = 1.21, α = 0.84).

#### Control variables

We decided a priori to control for three variables. The first was athlete gender, which is considered a major predictor of athletic performance, with men outperforming women^48^. The second variable was tenure with the coach. Although favorable impressions of narcissists wane with time^14^, we lacked longitudinal data to assess properly the relevance of coach impressions to athlete performance. The third variable was rowing distance. During the championships, most rowers perform individually and row 2000 m. First-year rowers, however, perform as a team (in individual heats) and may row slightly different distances, with better rowers rowing further (∼ 2100 m) than poorer rowers (∼ 1900 m). We controlled for this variable to avoid unnecessary noise.

#### Exploratory moderator: athlete narcissism

We were able to measure athlete narcissism for a subset of our sample (*n* = 215), across three years of the competition. This subsample included competitive rowers (50.2% women, 49.8% men; *M*_age_ = 21.14, *SD*_age_ = 4.25) from 31 rowing clubs, trained by 127 coaches (73.0% men, 27.0% women). Participants had, on average, 2.88 years (*SD* = 2.81) of rowing experience and had trained with their coach for 0.96 years (*SD* = 1.01). Athletes filled out the 40-item Narcissistic Personality Inventory (NPI-40^49^) and responded to the narcissism option from each of the original forced-choice items (e.g., “I want to amount to something in the eyes of the world”, “If I ruled the world it would be a much better place”, “I am an extraordinary person”) as either true or false (1 = *true*, 0 = *false*)^39^. We computed the NPI score as the average of the 40 items (*M* = 0.45, *SD* = 0.16, α = 0.82).

### Data analytic strategy

To take into account that athletes were part of different rowing clubs, and trained with different coaches, we ran multilevel analyses using a linear mixed model (also known as the random coefficient, hierarchical, or multilevel model^50,51^), including a random intercept. We used the Restricted Maximum Likelihood estimation, accounting for nesting within clubs and coaches. The linear mixed model is a well-established and powerful method of analyzing clustered data and addressing non-independence^52^. We conducted the analyses via SPSS statistical software, version 28. We applied the multilevel approach for both the confirmatory testing of our hypotheses and the exploratory moderation analysis. We calculated the total variance explained by the models with the marginal *R*^*2*^^53^. We centered and standardized all continuous predictors. The study data (excluding identifying information in accordance with EU data protection regulation) and syntax of analyses are available on OSF at: https://osf.io/8du2m/?view_only=467b032f79d64999a4e1103a4d912c91.

## Results

### Athlete performance and perceived self-enhancement opportunity

We present variable means, standard deviations, and correlations in Table 1. To test the coach narcissism and athlete performance (i.e., end time) link, we first entered the control variables (athlete gender, tenure with coach, rowing distance) as predictors (Table 2, Model 1). Adding coach narcissism as a predictor improved the controls-only model, χ^2^ = 8.54, *p* = 0.003 (Table 2, Model 2). We observed a negative link between coach narcissism and athlete end time, *B* =  − 3.52, *t*(236.66) =  − 2.94, *p* = 0.004, *r* = 0.19, 95% CI [− 5.88, − 1.16], which indicates a positive relation between coach narcissism and athlete performance.

**Table 1:** Means, standard deviations, correlations.

	*M*	*SD*	1	2	3	4	5	6
1. Athlete gender^a^	1.51	0.50						
2. Athlete age	21.34	4.49	0.04					
3. Distance (meters)	2003.39	26.22	− 0.10	− 0.02				
4. Tenure with coach (years)	1.01	1.20	0.03	0.30***	− 0.03			
5. Coach narcissism	0.44	0.23	− 0.17**	0.05	0.13*	0.01		
6. Athlete perceived self-enhancement opportunity	4.94	1.21	− 0.27***	− 0.02	0.18**	− 0.10	0.17**	
7. Athlete performance (end time)	7:07.86	0:35.73	0.81***	− 0.01	− 0.19**	− 0.12^†^	− 0.27***	− 0.33***

*N* = 257–266.

^a^1 = man, 2 = woman; Distance in meters; Tenure in years; Athlete end time in m:s.ss.

^†^*p* < .10, *p < .05, **p < .01, ***p < .001.

**Table 2:** Estimated coefficients examining the relationship between coach narcissism and athlete performance and perceived self-enhancement opportunity.

Fixed effects	Athlete performance (end time)						Athlete perceived self-enhancement opportunity					
	Model 1			Model 2			Model 3			Model 4		
	*B*	*SE*	*R*^*2*^	*B*	*SE*	*R*^*2*^	*B*	*SE*	*R*^*2*^	*B*	*SE*	*R*^*2*^
Constant	455.38	2.03		454.95	1.99		4.64	0.11		4.66	0.11	
Controls												
Athlete gender ^a^	57.48***	2.75		56.48***	2.72		− 0.63***	0.15		− 0.58***	0.15	
Distance	− 4.49***	1.11		− 3.98***	1.11		0.17*	0.08		0.15^†^	0.08	
Tenure with coach	− 3.75**	1.28	.680	− 3.74**	1.25		− 0.11	0.07	.102	− 0.11	0.07	
Predictor												
Coach narcissism				− 3.52**	1.20	.695				0.16*	0.07	.118
Random intercept (τ_00_)	224.93			203.23			0.09			0.09		
Model fit												
AIC	2245.00			2238.46			809.31			806.83		
BIC	2266.29			2263.31			830.60			831.67		
− 2LL	2233.00			2224.46			797.31			792.83		
χ^2^(1)				8.54**						4.48*		

*N* = 257.

^a^1 = man, 2 = woman; Distance in meters; Tenure in years; *R*^*2*^ = Marginal *R*^*2*^; Fit indices presented are based on ML estimation; χ^2^(1) is the difference between the − 2 log likelihoods of Model 1 vs. Model 2 and Model 3 vs. Model 4, respectively.

^†^*p* < .10, *p < .05, **p < .01, ***p < .001.

Using the same steps as above, we subsequently tested the relation between coach narcissism and athlete perceived self-enhancement opportunity. Adding coach narcissism again improved the controls-only model, χ^2^ = 4.48, *p* = 0.034 (Table 2, Model 4). We obtained a positive relation between coach narcissism and athlete perceived self-enhancement opportunity, *B* = 0.16, *t*(243.16) = 2.10, *p* = 0.036, *r* = 0.13, 95% CI [0.01, 0.30].

### Mediation analysis

We carried out the mediation model via the MLmed macro with Restricted Maximum Likelihood Estimation, including random intercepts. This macro allows for testing of 1-1-1 multilevel mediation models, while accounting for within-cluster and between-cluster variability, and estimates all of the parameters in the model simultaneously^54,55^. The macro estimates the indirect effect with a Monte-Carlo simulation that generates 95% confidence intervals using 10,000 resamples^56^. The Monte-Carlo method is recommended as viable for constructing confidence intervals for indirect effects in mediation analysis, because it helps to reduce the error rate and bias in multilevel mediation estimates, while being easy to implement and performing comparably to alternative methods such as bootstrapping^56–58^. The MLmed macro is suitable for testing 1-1-1 as well as 2-1-1 multilevel mediation models. Further, this macro has been reliably used in recent research involving multilevel mediation^59–64^, including research on narcissism^65–67^.

Given that the MLmed macro allows for one cluster variable, we controlled for the more proximate nesting within coaches (level two). The relation between coach narcissism and athlete performance was accounted for by athlete perceived self-enhancement opportunity, as the confidence intervals for the between-group indirect effect did not contain a zero, *B* = − 0.87, *SE* = 0.49, *Z* = − 1.77, *p* = 0.077, 95% CI [− 2.01, − 0.10]. The between-group indirect effect denotes that, across coaches, average athlete perceived self-enhancement opportunity accounts for the association between coach narcissism and average athlete performance^68^. Interestingly, the between-group direct effect between coach narcissism and athlete performance remained significant, *B* = − 4.75, *SE* = 1.65, *t*(154.33) =  − 2.87, *p* = 0.005, 95% CI [− 8.02, − 1.48], suggesting that additional mechanisms may be involved.

### Exploratory analysis: athlete narcissism as moderator

Given that we were able to measure athlete narcissism for a subset of our sample (*n* = 215), we explored whether narcissistic (vs. non-narcissistic) athletes are more strongly influenced by narcissistic coaches. The Coach Narcissism × Athlete Narcissism interaction was not significant either for athlete performance, *B* =  − 0.15, *t*(176.09) =  − 0.12, *p* = 0.907, 95% CI [− 2.60, 2.31], Random intercept (τ_00_) = 205.29, or athlete perceived self-enhancement opportunity, *B* =  − 0.02, *t*(198.22) =  − 0.29, *p* = 0.776, 95% CI [− 0.18, 0.13], Random intercept (τ_00_) = 0.12. Thus, athlete’s narcissism did not play a role in the influence of coach narcissism upon athletes’ performance.

## Discussion

Narcissism is prevalent among leaders. We examined whether and how coach narcissism is linked to athletes’ performance during competition, an opportune context for narcissistic coaches to show off their athletes’ performance and motivate them to display their skills and attain glory. Coach narcissism was positively associated with athletes’ performance, with rowers who trained with more narcissistic coaches evincing faster end times than those who trained with coaches scoring lower on narcissism. Moreover, athletes who trained with coaches higher on narcissism perceived the competition as an opportunity to self-enhance to a greater extent than athletes who trained with coaches lower on narcissism. Finally, athlete perceived self-enhancement opportunity, in turn, plausibly accounted for the positive relation between coach narcissism and athlete performance.

Narcissistic individuals are known to seek out and be motivated by competitive contexts, defined by high pressure, challenge, and audience presence, as these provide them with an ideal opportunity to self-enhance, showcase their skills, and attain personal glory^16,19^. Also, through vicarious self-enhancement^26^, narcissistic coaches can bask in the reflected glory of their winning athletes and, as such, would be similarly motivated to help them achieve superior performance during competition. Coaches can help raise athletes’ self-confidence through imagery of success and persuasion about athletes’ competencies^69^. Given that narcissistic individuals can be inspiring, charismatic, and persuasive^20,70^, these characteristics in a coach seem to be just what athletes need in competitive settings to spur on their motivation and performance as well as cope with competitive pressure, as our findings indicate. Future research could examine the type of verbal persuasion strategies that narcissistic coaches use and test how these strategies relate to athlete psychological resilience during competition, such as in their appraisal of competitive situations as challenges rather than threats^71^, and in turn athlete performance. Although our findings indicate that athletes who trained with narcissistic coaches perceived the competitive context as an opportunity to self-enhance, which in turn predicted better performance than for athletes who trained with coaches lower on narcissism, follow-up research may examine an alternative sequence whereby better performance begets more self-enhancement opportunity. It would be fruitful to find out how athletes rate the opportunity to self-enhance once they know how they performed.

The current research is the first to test directly the link between narcissism of coaches and the performance of athletes who train with them. Our findings mostly speak to the positive side of narcissistic coaches. This is in contrast to prior work on narcissistic coaches^11–13^ that reported evidence for the negative side of narcissists’ controlling coaching style. However, this positive/negative duality is consistent with literature on leadership, which considers narcissistic leaders to be a double-edged sword by simultaneously having a bright (e.g., charismatic vision) and a dark (e.g., aggression, exploitativeness) side^6^. Our findings suggest that, in competitive settings, the advantageous side of narcissistic leaders likely prevails. Athletes might be willing to overlook the negative characteristics of their narcissistic coaches in exchange for inspiration and confidence during competition. Indeed, in contexts of uncertainty, people choose narcissists as leaders, despite being aware of and acknowledging their negative characteristics, as people perceive that narcissistic leaders could reduce their uncertainty^15^. Stated otherwise, people seem to trade-off the narcissists’ negative characteristics for their positive ones.

Nonetheless, given narcissists’ many negative characteristics, such as their empathy deficits and controlling style, narcissistic coaches are unlikely to be functional across all contexts or for long. With the passage of time, they might instigate conflict and turmoil^6^. Moreover, narcissistic coaches might not be equally effective for all athletes. Athletes who are especially driven to succeed or talented might be more willing to be coached by a narcissistic individual who tries to maximize performance at all costs. On the other hand, athletes who are less achievement oriented or talented might be less tolerant of narcissistic coaching tactics and attitudes that prioritize results over other facets of athlete well-being, and these athletes might also suffer from greater coach-induced stress and pressure, which are likely to curtail their performance. Future research will do well to address the effectiveness of narcissistic coaches in non-competitive contexts, across time, and taking into account more thoroughly athlete individual differences (e.g., achievement motivation, current performance).

Although this study had several strengths, such as the inclusion of objective individual performance measures of athletes during competition and as a measure of their perceptions prior to the race, it also has limitations. First, due to its non-experimental design, we cannot ascertain causality. Nevertheless, given that we measured coach narcissism prior to the race, the influence is unlikely to operate in the opposite direction; that is, performance outcome is unlikely to influence ratings of coach narcissism. Follow-up investigations could employ longitudinal or experience sampling methodology designs to test the influence of coach narcissism in a more sequential manner over time. Second, it was not possible to obtain a baseline measure of athlete performance, which would have enabled us to check whether the improvement in performance during competition was greater for those athletes who trained with a more narcissistic coach. However, the absence of a baseline measure may be less problematic here given that the likelihood of a selection effect was low. As neither coaches nor athletes had discretion in whom they trained with, it was unlikely that narcissistic coaches would have attracted athletes who were better performers prior to the competition. Regardless, future research should include a baseline measure, if available. Third, we used other-rated narcissism rather than asking coaches to report on their own narcissism. This practice, though, has been successfully implemented in prior work^42^, with findings indicating that people are fairly accurate in detecting others’ narcissism^44^. Indeed, in this study, rowers with the same coaches were consistent in rating their coaches’ narcissism. Follow-up investigations could complement athlete’s perceptions of coach narcissism with coach self-reported narcissism. Fourth, our sample comprised participants from an individualistic culture, in which people are often encouraged to focus on themselves^72^. Athletes from individualistic cultures may be more readily influenced by narcissistic coaches in seeing competition as an arena in which to showcase their skills. Future research could test the replicability of our findings in collectivistic cultures where people would likely be encouraged to focus on the group. Finally, to extend the generalizability of our findings, follow-up work should consider testing athletes from other sports domains. For instance, narcissistic coaches may be particularly beneficial for individual-level competitions rather than team-level competitions, because self-enhancement opportunity predominantly concerns flaunting one’s individual achievement. Moreover, narcissistic coaches may be especially beneficial for endurance sports (e.g., rowing, swimming, cycling [time trials], running) rather than sports requiring more creative adaptation or strategy (e.g., football, basketball, tennis). Narcissistic coaches’ controlling coaching style might lend itself better to energizing athletes’ raw effort in more repetitive or mechanistic sports, while stifling their autonomous creative efforts.

To conclude, we demonstrated that coach narcissism positively predicts athletes’ performance during competition. Narcissistic coaches might do more good than harm when deployed by sports associations in some high pressure competitive contexts. These coaches might, for example, be particularly apt for giving athletes pep talks before the game or when stakes are high (e.g., in finals or during Olympics) to ensure that they perceive the competition as an opportunity to self-enhance and showcase their skills, thereby facilitating their performance. Our research makes a substantial contribution to literatures on narcissism in the sporting domain, narcissism in coaches, and, more generally, narcissism in leadership.

## Data Availability

The study data (excluding identifying information in accordance with EU data protection regulation) and syntax are available on OSF at: https://osf.io/8du2m/?view_only=467b032f79d64999a4e1103a4d912c91.
